# Participating in Physical Classes Using Eduball Stimulates Acquisition of Mathematical Knowledge and Skills by Primary School Students

**DOI:** 10.3389/fpsyg.2020.02194

**Published:** 2020-09-04

**Authors:** Ireneusz Cichy, Magdalena Kaczmarczyk, Sara Wawrzyniak, Agnieszka Kruszwicka, Tomasz Przybyla, Michal Klichowski, Andrzej Rokita

**Affiliations:** ^1^ Department of Team Sport Games, University School of Physical Education in Wrocław, Wrocław, Poland; ^2^ Faculty of Educational Studies, Adam Mickiewicz University, Poznan, Poland

**Keywords:** child development, learning, motor skills, physical activity, play

## Abstract

An increasing number of studies are evidencing relationships between physical activity (PA) and the mathematical performance of early school students. This is not surprising due to the fact that children grow in all areas simultaneously and their motor and intellectual developments determine each other. Nevertheless, such an approach of combining mathematics education with physical exercises, in addition through play, which is the basis of children’s activity and the preferred way of spending time, is still rare at schools. In response to this problem, “Eduball” has been created, which is an educational ball with printed letters, numbers, and other signs used for team mini-games. Surprisingly, despite the studies on general usefulness of Eduball in preschool and early-school education and the effects of physical exercise classes carried out using these balls, still little is known about their impact on mathematical development. Here, we investigate the relationships between the use of Eduball and the acquisition of mathematical knowledge and skills by children. We used a quantitative approach in the form of an experiment in natural settings in which 7-year-old students (first grade) took part (*N* = 25). For the purposes of this experiment, we created scenarios of physical exercise classes integrated with mathematical contents that used Eduball. Mathematical knowledge and skills were assessed by one of the commonly used tests. The results were compared with the data from the control group of traditional physical education classes (*N* = 22). As assumed, after a 1-year experiment, students from both groups improved their results, but we found a greater progress in terms of mathematical knowledge and skills in the experimental class compared to the control one. Eduball particularly affected competences related to such mathematical categories as: sets and their elements, multiplication and division, geometric shapes and measuring length, and measuring volume and mass. In sum, our results show that physical exercise classes that used Eduball stimulate the acquisition of mathematical competences by students and, consequently, confirm that there is a strong relation of physical and mathematical development. Therefore, there is a need to review children’s educational models, as well as primary school curricula, to combine physical and cognitive activities.

## Introduction

It is well-established that physical activity (PA) during education time can be beneficial for children’s academic performance (Beck et al., 2016; Donnelly et al., 2016; Watson et al., 2017; Mullender-Wijnsma et al., 2019; Norris et al., 2019; Vaquero-Solis et al., 2020). Moreover, it was recognized that schools should find more opportunities for increased PA within the elementary school day for many health benefits (Mendo-Lazaro et al., 2017; Bartholomew et al., 2018). This new way of teaching – physically active lessons (PAL), in which physical exercises are integrated into academic lessons – may provide an opportunity to increase school-based PA while concurrently having a positive impact on academic-related outcomes (Watson et al., 2017). Mathematics is the mother of all sciences, and the world cannot exist without it; also, in school reality, it is the core of education, and mathematics is a compulsory subject worldwide. Nevertheless, there are a number of children who do not like mathematics, do not like learning this subject, and find it difficult to learn. Therefore, it is so important to foster acquisition of mathematical knowledge and skills. Integrating PAL with mathematical contents in the classroom is a newly explored approach, and according to the latest studies, it may enhance children’s mathematics performance (Beck et al., 2016; Mullender-Wijnsma et al., 2016; Vazou and Skrade, 2017; Sneck et al., 2019; Vetter et al., 2019), as well as increase children’s academic intrinsic motivation (Vazou et al., 2012). Additionally, motor enriched learning activities can improve the academic achievement of children from disadvantaged group (Mullender-Wijnsma et al., 2019). There are also evidences which suggest that classes with exercises on gross motor skills led to larger improvements than exercises on fine motor skills, but it is not the case for low math performers (Beck et al., 2016). To sum up, PAL is a promising new way of teaching mathematics but the question is how to best incorporate PA into schools.

The basic form of physical exercise in early school education (and of all children’s activity) is play. It is a preferred way of spending time for children, and it is a natural way to meet children’s interests and motor, cognitive, emotional, and social needs. Through play, children learn basic skills such as addition, subtraction, calculation, and classification, since informal reasoning represents a foundation on which formal mathematics can be construed (Shaklee et al., 2008). Piaget (1951) stressed that understanding mathematics stems from children’s activity rather than the formal teaching of math (see also Tudge and Doucet, 2004). These views were supported by Sarama and Clements (2004), who argued that everyday children’s experiences represent a foundation for mathematics. The study by Marcon (1992) demonstrated that the greatest progress, especially in mathematics, occurs when activities are taken by children themselves. Therefore, a number of educators use play as a tool for teaching mathematics in groups of small children. Such students need to hear language, rhymes, and sounds of early alphabetization and need various experiences in order to develop numeral minds (Shaklee et al., 2008). Children can learn mathematics during an informal play, but rich and distinct analysis of mathematical relationships is possible only with the supervision of adults. Early mathematics is a very broad concept, and informal play does not always support mathematization, interpretation of mathematical experiences, and understanding of the relationship between each other (Lee and Ginsburg, 2009). On the other hand, the observation of children and participation in play initiated by children helps to develop their mathematical thinking (Doverborg, 2006). Teachers often use games and play in the process of early mathematization using such aids as blocks, jetton, and colorful boards. However, they rarely use the opportunity of connecting the contents of curricula, such as linking PA with cognitive activity through motor play.

**Figure 1:**
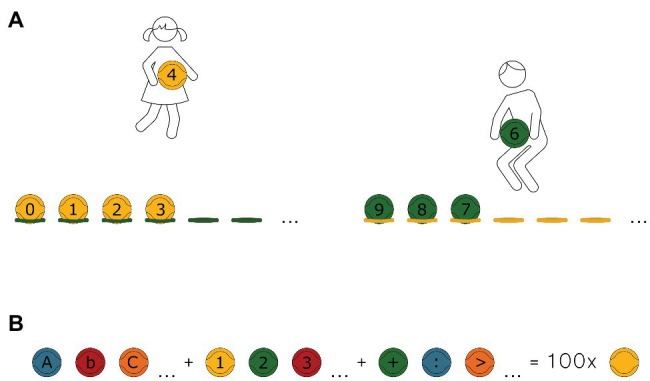
Eduball educational balls. **(A)** Example of an educational game: “From 0 to 9.” The students, divided into two teams (yellow and green), freely move across the whole gymnasium. At the teacher’s signal, the students with the yellow balls need to line up as fast as they can from smallest to biggest number. The green team has to line up from biggest to smallest number. **(B)** Eduball educational balls means 100 balls used for mini-games for teams in five colors (yellow, green, blue, red, and orange) with painted (in black) letters of alphabet (large and small letters), digits from 1 to 9 and 0, mathematical operation symbols [addition (+), subtraction (−), multiplication (×), division (/), greater than (>), smaller than (<), and brackets ()] and symbols of the electronic mail (@). Placing letters, numbers, and characters on the balls allowed for their comprehensive use in various areas of science during physical activities.

It should be noted that early school age (period of early childhood) is critical to children’s development – they have to acquire a number of skills (cognitive, communication, and movement-related). Social, physical, emotional, and intellectual developments are very intensive at this age. It should be remembered that these components, although independent to a certain degree, are exposed to various interactions. For instance, motor development is, in many aspects, interrelated with emotional, intellectual, or social development. According to Woodfield (2004), children develop and grow in all physical, emotional, social, linguistic, intellectual, and creative areas simultaneously. Education should occur with regard to these mutual relationships. What we do for motor development is not merely an investment in health or the formation of abilities: it is an investment in intellectual, social, and emotional development. These mutual relations and willingness to offer children an opportunity to learn through motor play were the inspiration for creation of “Eduball” educational balls. In brief, Eduball is one of the latest methodical proposals combining PA with integrated subject content. In this interdisciplinary teaching approach, an innovative didactic aid is used in the form of a set of balls (dedicated to team games) on which letters, numbers, and other characters are printed. With Eduballs, teachers can stimulate students in different development areas at the same time and students can learn by movement, all in a positive atmosphere of games and play. The idea of such balls was born in Poland in 2002. Since then, Eduball has been studied and developed in many countries around the world (Rokita et al., 2017a,b, 2018), which did not cause any problems because education in Poland (taking into account such aspects as the average class size, the goals of education, curriculum, and instruction time per subject) does not differ significantly from most Western countries (see OECD, 2019). Currently, Eduballs are used in several 100 schools in Poland and in other European countries, as well as in the United States. There is no curriculum of Eduball-class – it should naturally merge with academic learning. Teachers can use a book with examples of games using Eduballs (e.g., Rokita et al., 2017b), but this is not a closed collection. Ideally, they should create their own solutions for adjusting the activities to the currently studied didactic material. Figure 1A shows an example of an educational game with Eduballs called “From 0 to 9” (in which at the teacher’s signal, students divided into two teams and need to line up as fast as they can from smallest to biggest number and conversely; see Supplementary Material for more examples), and Figure 1B demonstrates elements of the Eduball set.

Our previous studies (e.g., Rokita and Rzepa, 2000, 2002, 2003, 2005; Rzepa and Wojcik, 2007a,b,c,d; Rokita, 2008; Cichy et al., 2010; Krysmann and Rokita, 2011; Rokita and Kaczmarczyk, 2011; Rokita and Cichy, 2012, 2014) show that physical exercise classes (integrated with subject-related contents) that use Eduball help to develop various skills. For example, we found that while using these balls during physical exercise classes, children are involved in various social relations, which form social behaviors (e.g., cooperation, decision skills, or communication skills). Such – offered by Eduball – connecting PA with intellectual activity in the process of teaching and physical education motivates children to participate in activities and falls within a holistic approach to implementation of curricula and assumptions of integration around physical education. In addition, no deterioration in physical fitness (and even the opposite tendency) was observed in any previous Eduball-experiments. As for cognitive effects, we have mainly studied writing and reading skills and we did not pay too much attention to mathematical skills. What is more, most of our reports were published in Polish. Thus, it is necessary to explore the critical issue of Eduball – the integration of physical exercises (conducted with Eduball) with mathematical contents – and present the results of these investigations to an international group of researchers.

All in all, there is a general agreement that combining PA with cognitive activity is beneficial for academic performance, and play is an excellent way of learning for children in the first stage of education. Eduball is such an approach to teaching that fosters acquisition of various skills and knowledge. Moreover, recent research indicates that integrating PAL with mathematical content may have a positive effect on mathematical education results, but on the other hand, still little is known how to put this concept into practice. Eduball may be a remedy for this challenge; therefore, it should be examined whether this is the case. Thus, the aim of this study was to investigate whether or not teaching physical exercise classes using Eduball as a supplementary resource would cause substantial changes in the mathematical knowledge and skills of primary school students. We hypothesize that using Eduball is beneficial (and more effective than traditional methods) in the acquisition of mathematical knowledge and skills in such a group. The outcomes of our 1-year experiment confirm this hypothesize, as well as shed a new light on strategies of integrating PAL with mathematical content in natural settings such as school.

## Materials and Methods

### Participants

Forty-seven Polish 7-year-old first-grade students participated in the experiment. The children were randomly divided into two groups: the experimental class (*N* = 25, 12 girls) and the control class (*N* = 22, 9 girls). There was no any attrition with the 1-year follow-up in either group – all students participated in the first and the second examination, and their attendance was regular/normal. To be sure that the control and experimental groups are homogeneous, the tests of intellectual abilities and physical fitness were also carried out (e.g., International Test of Physical Fitness, Pilicz et al., 2004). No significant differences were found between the groups (see Supplementary Table 1 for more details).

### Procedure

Our study was assessed and approved by the local Ethics Committee for Research Involving Human Subjects (Resolution of the Senate Committee on Ethics of Scientific Research at the University School of Physical Education in Wroclaw of November 20, 2009). As such, all procedures and manipulations were carried out in accordance with the principles of the Declaration of Helsinki.

**Figure 2:**
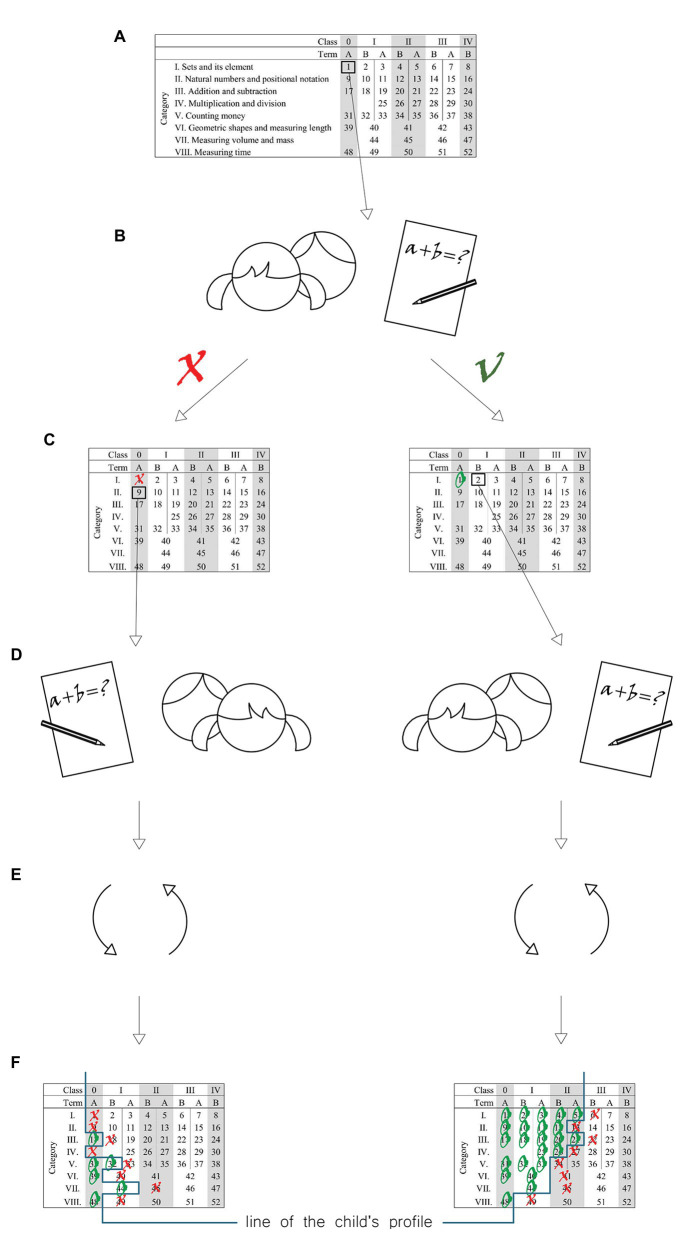
Structure of the mathematical test procedure. **(A)** The child begins to take the test from the first category task at the zero (0) level. **(B)** Each time the child solves a task, the task is marked in the plane with a circle, whereas an improperly solved task is crossed out. **(C,D)** If the task is solved properly, the child is FIGURE 2asked to solve another task (more difficult) in the category. **(E)** This procedure should be repeated until the task turns out to be too difficult: this helps to determine the upper limit of the child’s abilities to independently solve the task. In the case of the task not solved in the primary series, the child solves the task in the next category. **(F)** After the result of the independent work of the child is marked in the test plan, the profile of child’s abilities to solve tasks independently is determined (the line of the profile runs exactly between the circles and crosses).

The experiment was carried out in natural conditions using the technique of parallel groups as it was the basic tool for investigating the cause-and-effect relationships. An experimental factor was the author’s program of physical exercise classes taught using Eduball. In the experimental class, two of three 45-min physical exercise classes a week were carried out using Eduball. In the control class, all three 45-min physical exercise classes were taught without using educational balls. The scenarios for physical exercise classes using Eduball were developed together with the home room teacher. The classes were integrated with mathematical contents according to the cycle and topics for individual days. Physical exercise classes were taught by home room teachers from individual classes. The dependent variables were represented by the mathematical knowledge and skills of students. Physical fitness was not regarded as a dependent variable since it was diagnosed in order to examine group homogeneity (Examination 1) and in order to demonstrate that the use of Eduball in physical exercise classes did not cause a regression in the physical fitness of students (Examination 1 vs. Examination 2). The pre-test and post-test examinations, carried out at the beginning and the end of the school year (which in Poland begins in September and ends in June), included the diagnosis of mathematical knowledge and skills, as proposed in the test by Gruszczyk-Kolczynska et al. (1985). This Polish math test was used because it is standardized to Polish educational conditions. According to the assumptions of the test, eight categories were used during the evaluation of mathematical knowledge and skills: sets and elements of sets, natural numbers and positional notation, addition and subtraction, multiplication and division, counting money, geometric shapes and measuring length, measuring volume and mass, and measuring time. The diagnosis was based on the evaluation of the level of mathematical knowledge and skills and relies on independent solving of mathematical tasks by children. This test contained 52 scenarios which were divided into eight above-mentioned categories. The basis for construction of the test was the plan of test in a form of matrix (see Figure 2A). The matrix includes all the tasks that evaluate the level of mathematical knowledge and skills of zero-, first-, second-, and third-grade students at the end of winter and summer terms. The eight columns in the matrix correspond to individual categories, whereas the rows represent individual years of mathematical education. Empty fields mean that implementation of the contents in the area of a specific category begins later. Numbers from 1 to 52 mean individual tasks. The tasks in the same categories are arranged from the simplest to more complex. Figure 2 shows the scheme of this mathematical test procedure.

### Data Analysis

Despite the fact that we ran both qualitative analysis (for ability profiles, see Figure 2F) and quantitative analysis (for numbers of solved tasks), we present here only the results of the main (quantitative) procedure. We used mean and standard deviation (SD) as descriptive characteristics of the results obtained from the examination of the students from control and experimental groups, also with consideration of genders. Comparison of mean results obtained by students in the first and the second examination in the experimental and control group was made by means of the non-parametric Mann–Whitney U-test. The significance of differences in the results (increments of the variable studied during the experiment) of students both in the control and experimental classes was analyzed using non-parametric Wilcoxon Z-test. This test was also used in order to determine differences in the results obtained by boys and girls from both classes. The adopted level of significance was *α* = 0.05. All statistical analyses were carried out using Statistica 13.0 (Dell, Round Rock, Texas, United States).

## Results

**Figure 3:**
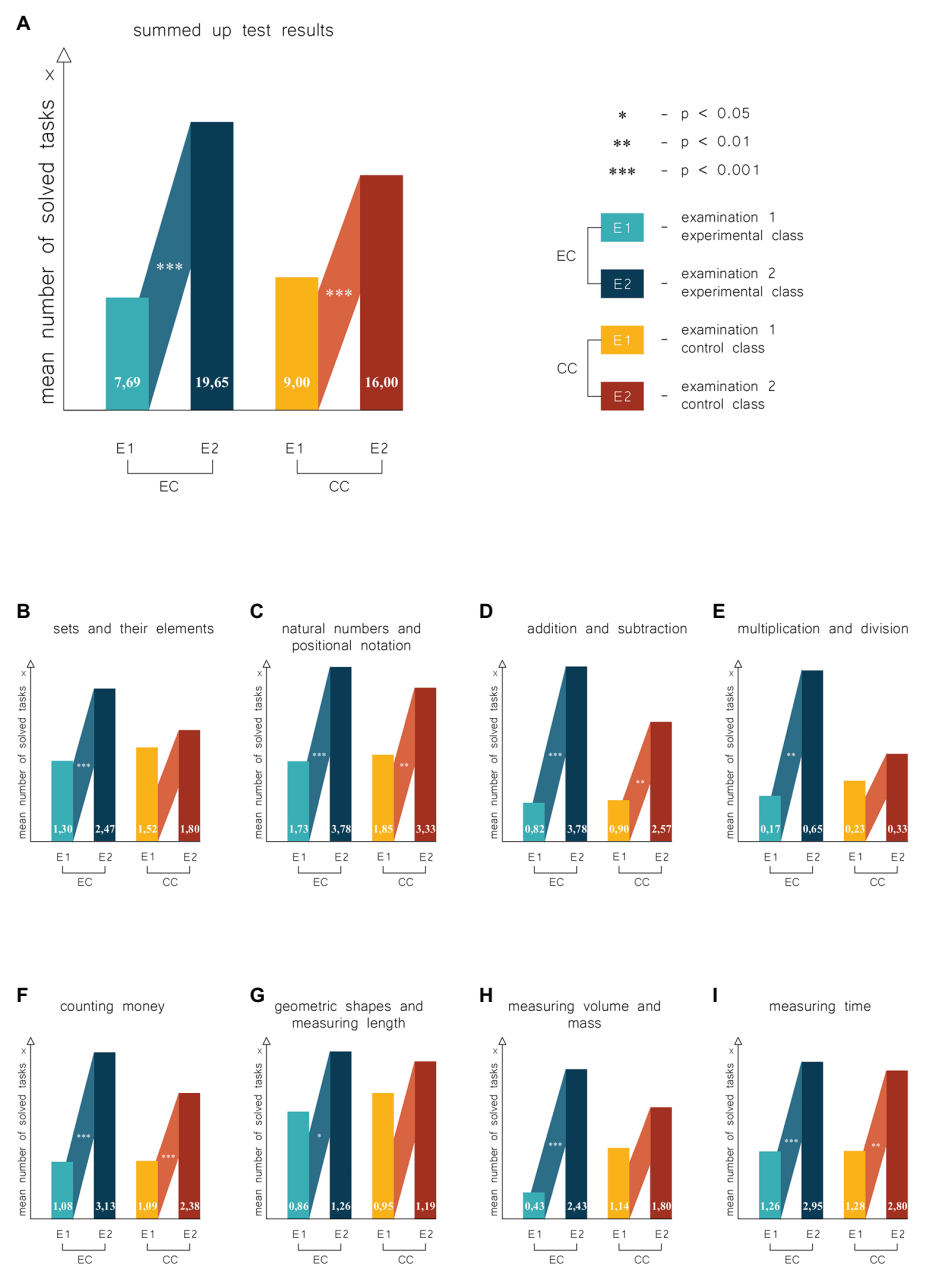
Results of experiment. Statistical analysis of the results in terms of mathematical knowledge and skills included eight categories consistent with the assumptions of the mathematical diagnosis test. **(A)** Results of experiment for all categories. **(B)** Results for the category: sets and elements of sets. **(C)** Results for the category: natural numbers and positional notation. **(D)** Results for the category: addition and subtraction. **(E)** Results for the category: multiplication and division. **(F)** Results for the category: counting money. **(G)** Results for the category: geometric shapes and measuring length. **(H)** Results for the category: measuring volume and mass. **(I)** Results for the category: measuring time. EC, experimental class; CC, control class; Examination 1, the examination at the beginning of the school year (in September); Examination 2, the examination carried out at the end of the school year (in June).

As Figure 3A shows, at the beginning of the school year, students from the experimental class solved, on average, one task fewer than their peers from the control group. The situation at the end of the school year was changed: students from the experimental group solved, on average, several tasks more than the students from the control group; mean results obtained by the students during the Examination 1 were 7.69 (*SD* = 5.02) in the experimental class and 9.00 (*SD* = 5.61) in the control class, and 19.65 (*SD* = 7.81) in the experimental class and 16.00 (*SD* = 6.93) in the control class during the Examination 2. Both students from the experimental class and students from the control class significantly improved their results after the school year [for experimental group *Z* = 4.20, *r*-based effect size (*r*) = 0.61, and for control one *Z* = 4.00, *r* = 0.58, both *p* < 0.001].

The first task category analyzed was “Sets and their elements.” At the beginning of the school year, students from the experimental class obtained the mean of 1.30 (*SD* = 0.70) in tasks concerning sets and their elements, whereas the students from the control group obtained the mean of 1.52 (*SD* = 0.51); mean results of students from these classes during the second examination were 2.47 (*SD* = 0.59) and 1.80 (*SD* = 0.68; Figure 3B). Differences between the mean results obtained by the students from the experimental and control classes in the first examination were not statistically significant (*p* > 0.05), contrary to the second examination, where a significant difference was observed in favor of the experimental class (*U* = 120.50, *p* < 0.001, *r* = 0.48). As Figure 3B shows, the comparison of the results obtained by the students from the experimental class at the beginning and at the end of the school year revealed significant differences. Each student from the experimental class solved at the end of the school year one task more than at the beginning of the year (and this was a significant difference as such *Z* = 3.82, *p* < 0.001 and *r* = 0.56). Students from the control class improved their results on average by 0.28, which does not represent a statistically significant difference (*Z* = 1.89, *p* = 0.06, *r* = 0.28).

In the category of “Natural numbers and positional notation,” both in the first and second examination, no significant differences were found between the mean results obtained by students from both classes (both *p* > 0.05). As Figure 3C shows, students from the experimental class obtained the mean of 1.73 (*SD* = 0.91) in the first examination and 3.78 (*SD* = 1.20) in the second, whereas the results in the control groups were 1.85 (*SD* = 1.31) and 3.33 (*SD* = 1.28), respectively. Therefore, students from the experimental class solved at the end of the school year, on average, two tasks more than at the beginning of the year (the difference between the means was 2.05). An insignificantly smaller progress was observed in the students from the control class (the difference in means was 1.48). The differences in the increase of knowledge and skills in the category studied are statistically significant in both cases (for the experimental group *Z* = 4.01, *p* < 0.001 and *r* = 0.59, and for control one *Z* = 3.61, *p* < 0.01 and *r* = 0.53).

In solving tasks in category “Addition and subtraction,” students from both classes obtained similar results at the beginning and the end of the school year [means obtained for the experimental class were 0.82 (*SD* = 1.15) and 3.0 (*SD* = 1.86), whereas students from the control class obtained 0.9 (*SD* = 1.41) and 2.57 (*SD* = 1.94), respectively, all *p* > 0.05]. Students from both classes solved, on average, one task during the Examination 1, whereas in the Examination 2, almost all students solved three tasks (Figure 3D). Students from the control and experimental classes obtained similar results in both examinations. Therefore, it is difficult to conclude whether either of classes was better. Students from both classes solved, on average, two tasks more at the end of the school year, thus improving their results. The differences between the results at beginning and at the end of the school year in this category were statistically significant in both classes (for the experimental group *Z* = 4.01, *p* < 0.001, *r* = 0.59, and for control one *Z* = 3.16, *p* < 0.01, *r* = 0.46).

Fourth category “Multiplication and division” represents a specific class. When the test we used was published, the diagnosis of multiplication and division skills began from the summer semester of the first year, because, at that time, learning of multiplication and division skills began in Polish schools. At present, these skills are taught in the second grade. Although children attempted to solve tasks in Category 4, these attempts were rarely successful i.e., the tasks were rarely solved. Therefore, the mean results obtained by the children were at a low level. At the beginning of the school year, each student solved one task on average, and the mean result obtained by pupils in the experimental class was 0.17 (*SD* = 0.65), whereas this value in the control class was 0.23 (*SD* = 0.44; Figure 3E). At the end of the school year, situation in the control class almost did not change (the mean = 0.33, *SD* = 0.73, *p* > 0.05), whereas in the experimental group, the mean increased to 0.65 (*SD* = 0.88), which, although not being a substantial difference between the results obtained in the classes (*p* > 0.05), is a significant progress (*Z* = 2.93, *p* < 0.01, *r* = 0.43).

In the first examination of category “Counting money,” students from both classes obtained almost identical mean of the results [1.08 (*SD* = 0.79) in the experimental class and 1.09 (*SD* = 0.83) in the control class], each student solved one task on average. Furthermore, as Figure 3F shows, in the second examination, the mean result from the experimental class increased to 3.1 (*SD* = 1.25), whereas in the control class, this value rose to 2.38 (*SD* = 1.43). Differences between the results obtained in these classes were not statistically significant in both examinations (both *p* > 0.05). The comparison of the results obtained at the beginning and at the end of the school year shows that students from both classes made a substantial progress over the school year. The differences turned out to be statistically significant (for the experimental group *Z* = 4.11, *p* < 0.001, *r* = 0.60, and for control one *Z* = 3.34, *p* < 0.001, *r* = 0.49).

As Figure 3G shows, in category “Geometric shapes and measuring length,” students in the experimental group obtained, in Examination 1, the mean of tasks solved of 0.87 (*SD* = 0.34), whereas this value in Examination 2 was 1.26 (*SD* = 0.69). The students from the control class obtained means of 0.95 (*SD* = 0.22) and 1.19 (*SD* = 0.60), respectively. Differences between the results obtained by the students from both classes at the beginning and at the end of the school year were not statistically significant (both *p* > 0.05). Students from the experimental class improved (statistically significantly) the results over the school year (*Z* = 2.02, *p* = 0.04, *r* = 0.29). Their peers from the control class failed to improve the results (*p* > 0.05). The tasks rely on geometric shapes and measuring length turned out to be exceptionally difficult for the students. Although the first (the easiest) task from this category was solved by almost all the students, another one (slightly more difficult) was solved at the end of the school year by only two students from the control class and three from the experimental class.

“Measuring volume and mass” is a category where no tasks were planned for the first grade in the first term (similar to multiplication and division). However (see Figure 3H), in the first examination carried out in the experimental class, every second pupil, on average, solved one task expected to be solved in the second term (the mean = 0.43, *SD* = 1.04), whereas in the second examination, the mean was 2.43 (*SD* = 1.34). Mean results in the control class at the beginning and at the end of the school year were 1.14 (*SD* = 1.36) and 1.80 (*SD* = 1.40), respectively. The students from the experimental class obtained worse results than their peers from the control class at the beginning of the school year (but this difference was not statistically significant, *p* > 0.05) and better results at the end of the school year, although these results were also not statistically significant (*p* > 0.05). Comparison of the results of the first examination with the second revealed that the students from the experimental class improved their results significantly (*Z* = 3.61, *p* < 0.001, *r* = 0.53), contrary to the students, from the control class, who did not improve their results (*p* > 0.05).

In the last category “Measuring time” (see Figure 3I), students from the experimental class obtained at the beginning of the school year the mean of 1.26 (*SD* = 1.18), whereas this value in the control class was 1.28 (*SD* = 1.10). In the second examination, these means were 2.95 (*SD* = 1.84) and 2.80 (*SD* = 2.18), respectively. Differences between mean results obtained in these classes were not statistically significant in both examinations (all *p* > 0.05), and students from both classes improved their results significantly during the experiment (for the experimental group *Z* = 3.82, *p* < 0.001 and *r* = 0.56, and for control one *Z* = 3.14, *p* < 0.01 and *r* = 0.46).

For all analyses, no significant differences were found in terms of genders. Furthermore, comparing the pre‐ and post-test of physical fitness, no deterioration was observed in this area. These additional results show that the use of Eduball in physical exercise classes is effective for both boys and girls and also that it does not cause a regression in the physical fitness of students.

## Discussion

Our study shows that participating in physical classes using Eduball causes a faster acquisition of mathematical skills, as well as knowledge. This effect might result from the specificity of playing with such educational balls, how they can be used, and what distinguishes this approach to learning from other games is combining physical and cognitive activities. Although there was a general progress in both studied groups, only participants from the experimental class improved their results significantly in all mathematical categories which we analyzed. Students from the control class improved their results only in four of eight categories (strongly related to operations on numbers such as addition, subtraction, counting money, and measuring time). What distinguishes the experimental group is the fact that, in its case, there was also an improvement in the mathematical imagination (multiplication and division) and spatial imagination (sets and their elements, geometric shapes and measuring length, and measuring volume and mass), which are not so closely related to numbers. These are definitely more abstract activities that require a different mathematical thinking. Such an observation shows that learning with Eduball creates optimal conditions for holistic mathematical development. Therefore, our findings have a very important implication: since there is such a strong impact of PA on the acquisition of diverse mathematical knowledge and skills by students, there is a need of teaching mathematics using games and plays based on PA, as well as using the idea of PAL. Educational balls such as Eduball are one of the tools/methods that make this possible.

Many previous studies postulate a necessity of teaching mathematics through game and play which utilize the natural spontaneity of child. Zammarelli and Bolton (1977) carried out a study using play in mathematical education. After the examination of 24 children aged 10–12, they found that the use of play in teaching mathematics is conducive to improved understanding of this subject. Similar conclusions were drawn by Rogers and Miller (1984). These researchers had their first-grade students solve a test that consist of multiplication and multiples tasks. The students who played before solving the tests achieved better results than those who were not involved in games and play before solving the test. The authors concluded that the properly designed play is conducive to learning mathematics. Furthermore, Pellegrini (2005) found that children who play at breaks return to classes more motivated for work. The view which says that play supports the development of positive basics of mathematics in learners was expressed also by Herringer. This researcher suggested that teaching mathematics using play is conducive to development of self-confidence and motivates for mathematical activity (Hirsh-Pasek et al., 2009). Moreover, the experiment carried out by Seo and Ginsburg showed that 4‐ and 5-year-old children acquire basic mathematical concepts during play. Regardless of the social class of the children, three categories of mathematical activity were broadly spread: playing with patterns and shapes (recognition of patterns and spatial forms), playing with evaluation of the size (comparing the size or comparing two or more objects in order to evaluate the relative size), and playing with counting (qualitative or quantitative evaluation; Hirsh-Pasek et al., 2009).

It seems that teachers should implement games and play in mathematical education. Spontaneous play might contain direct mathematical contents and small children might play with discovering numbers and shapes. Children learn mathematical relations every day and use mathematics as they play (Singer et al., 2006). It often seems that, as Kagan and Lowenstein (2004) argued, the term “children’s play” sometimes sounds as an oxymoron. Learning in school is often associated with the necessity of sitting at the desks, re-writing from the blackboard, which, to the children at this age, is just boring. Their activity, so natural in the cognitive development, is limited at school to sitting at the desks (Whitehurst, 2001). But through play, children can learn vocabulary, concepts, solve problems, develop memory and creativity and form language abilities (Hughes, 1999; Singer et al., 2003; Hirsh-Pasek et al., 2009) or skills of reading and writing (Christie, 1998; Owocki, 1999). When playing, children can also acquire mathematical skills (Seo and Ginsburg, 2004; Ginsburg, 2009). Both play and mathematics are inherent in children’s activity from the youngest age. Despite their immaturity, small children think mathematically, ask mathematical questions, look for solutions, and use mathematics to solve actual problems. Play represents a promising background for teaching mathematics. The problem is how to teach children mathematics in a way that is consistent with both curricula and everyday children’s mathematical activity? This leads to the conclusion that curricula should contain a number of elements of play in order to maintain a natural enthusiasm which characterizes children and their everyday activity. The curricula should also include broadly-understood mathematics rather than be limited to concrete tasks. It is known that everyday child’s activity can engage abstract concepts. The curricula should be “full of play” (Ginsburg, 2009).

In order to use mathematical types of play in schools, it is necessary to find the connections between theoretical ideas and practical solutions. Firstly, inclusion of mathematical play in teaching mathematics necessitates the support from the school environment. Introduction of mathematical play causes the need of discovering not only the mathematical phenomenon but also new materials. The role of the teacher is also very important as teachers are able to inspire for play and learning mathematics at school (Klerlein, 2006). Van Oers (1996) notes that the potential of specific play to make mathematical thinking easier depends largely on teacher’s skills to use the opportunities appropriately. This ability is guaranteed by having mathematical knowledge, understanding the nature of children’s play, with particular focus on the play which promotes learning mathematical thinking and awareness of the role of adults in promotion of both play and mathematical reasoning. Home-room teachers, who are sensitive to these problems and can teach mathematics during play, might contribute to better understanding of mathematics. Teachers might create children’s disposition for play, curiosity, critical, and creative thinking (Carr, 2001).

The question arises when it can be expected that the use of mathematical games and plays in teaching mathematics make teaching this subject more effective? Although game and play might not guarantee mathematical development, it undoubtedly offers a number of opportunities, particularly when teachers perceive these opportunities, attempt to respond to them, and present mathematical concepts during the play. Our study shows that the selection of appropriate games and plays, and linking them to PA, might be the key here. It is not surprising as neurosciences show that mathematical skills are rooted in bodily experiences, which is called the idea of embodied math representations (Domahs et al., 2010). Neuroscientists point out that, for example, because we learn (as children) to count using our fingers, mathematical cognition is related to representations of hands in our brain, or even representations of a fine motor serve as a scaffolding for math knowledge (Marghetis and Nunez, 2013; Riemer et al., 2016; Klichowski and Kroliczak, 2017; Klichowski and Przybyla, 2017; Rugani et al., 2017). Thus, stimulating young children with various hand games leads them to improving not only their motor fitness but also fosters children’s numerical performance (Gracia-Bafalluy and Noel, 2008). The neural link between math and manual skills is, thus, not only concerned with the fact that these two functions utilize the same (or closely related) anatomical structures (Friedrich and Friederici, 2013) but also with the process of acquiring mathematical competences. From the perspective of neurosciences, a dexterity training becomes an important element of the process of teaching young children mathematics. Our results show that such a relation also applies to a gross motor training of primary school students. Future research is needed to explore and extend these promising observations. It should be carried out with the use of research tools measuring not only behavioral effects, as in our study, but also with techniques such as neuroimaging or other methods in the field of cognitive neuroscience. Such research may not only enable – as in the case of our study – showing the practical consequences of using educational balls but also develop a theory concerning the relationship between gross motor activity and mathematical cognition, which is still not so well studied (Ras et al., 2019; Klichowski et al., 2020; Klichowski and Kroliczak, 2020). Without a doubt, it is also necessary to conduct further, more thorough studies into Eduball’s influence on children’s mathematical development that will involve participants of other age and cultural groups and use other experimental procedures, as well as skill tests. Another challenge could be to conduct a research in other countries (note that children’s education system in Poland operates in a very similar way to that in most Western countries, so it is worth testing Eduball’s effectiveness in other realities with different educational conditions).

## Conclusion

This study indicates that participating in physical classes using Eduball might cause a faster acquisition of mathematical knowledge and skills. Children from the experimental class improved their results significantly in all mathematical categories tested here (sets and their elements, natural numbers and positional notation, addition and subtraction, multiplication and division, counting money, geometric shapes and measuring length, measuring volume and mass, and measuring time). Such an effect was not observed on the students from the control class, who improved their results only in four of these categories (natural numbers and positional notation, addition and subtraction, counting money, and measuring time). These changes were furthermore lower than in the experimental group. Thus, the recommendation to use educational balls such as Eduball for teaching mathematical contents integrated with physical exercise classes.

## Data Availability Statement

The raw data supporting the conclusions of this article will be made available by the authors, without undue reservation.

## Ethics Statement

The studies involving human participants were reviewed and approved by Ethics Committee for Research Involving Human Subjects (Resolution of the Senate Committee on Ethics of Scientific Research at the University School of Physical Education in Wroclaw of November 20, 2009). Written informed consent to participate in this study was provided by the participants’ legal guardian/next of kin.

## Author Contributions

This project was conceptualized by AR, IC, and MKa. Data were collected by MKa, analyzed by IC, AR and MKa, and interpreted by IC, MKa, AR, AK, and MKl. The manuscript was written by all the authors. Figures were prepared by AK. All authors contributed to the article and approved the submitted version.

### Conflict of Interest

The authors declare that the research was conducted in the absence of any commercial or financial relationships that could be construed as a potential conflict of interest.
