# Supplementary material for: Participating in Physical Classes Using Eduball Stimulates Acquisition of Mathematical Knowledge and Skills by Primary School Students
Source: Front Psychol. 2020 Sep 4;11:2194. doi: 10.3389/fpsyg.2020.02194 (PMC7498696; doi:10.3389/fpsyg.2020.02194)
Supplement: Supplementary file 1 [file Data_Sheet_1.pdf]

Supplementary Material

1. Examples of Eduball games[1]

1.1. Mathematics Education

1.1.1. Game: 4 Tasks

■ Objective: to get familiar with the digit "4", improve the ability to work in a team, and practice certain motor skills.
■ Number of participants: any number
■ Props: Eduballs
■ Area: gymnasium or open air
■ Description: the teacher says "4" aloud. At the center of the pitch, the students should form the digit "4" out of their bodies. The students then have to complete four tasks as quickly as possible and return to their starting position. The following are the tasks:
■ Task 1: divide the balls according to their colors and put them into four corners of the gymnasium.
■ Task 2: collect the balls with the digit "4".
■ Task 3: each student should gather any four balls.
■ Task 4: each student should say four words beginning with the letters on the collected balls.

1.1.2. Game: Attractive Digits

■ Objective: to improve the students' knowledge of the digit order in an increasing number sequence, practice their perceptiveness and orientation in space and time, and improve certain motor abilities and physical skills.
■ Number of participants: any number
■ Props: an Eduball for each participant
■ Area: gymnasium or open air
■ Description: each student has a ball with a digit from 0 to 9. They move about in different directions. At the teacher's signal, they have to pair up with someone who has the successor, e.g. 12, 67. After that, the students move in pairs. At the next signal, the students should form groups of four (e.g. 6789) or three (e.g. 123). The game is over when the students have formed the entire number sequence from 0 to 9. The teacher gives instructions as to how the students should move across the pitch.

1.1.3. Game: Compare Sets

■ Objective: to practice the use of mathematical signs (<, >, =) and mathematical operations, develop the ability to work in a team, and improve certain motor skills.
■ Number of participants: any number
■ Props: Eduballs, hoops and rubber rings
■ Area: gymnasium or open air

---

[1] Based on our book: Rokita, A., Cichy, I., Wawrzyniak, S. and Korbecki, M. (2017). *Eduball Games and Sports*. Wroclaw: AWF.

■ Description: the students are divided into five teams. Two hoops and a rubber ring between these hoops are assigned to each group. Eduballs are strewn across the pitch. The first task of each team is to place green and yellow balls into the hoops within 30 seconds. The next task is to count how many balls there are in each set and to place a ball with a sign (<, =, >) accordingly on the rubber ring. After the task has been completed, the whole team has to run around their sets three times as fast as they can and return to their starting/finishing position. After that, the teacher checks the correctness of the comparisons.
■ Variation: a more difficult task would be to sum up the numbers on the balls in the hoops and placing the relevant sign (<, >, =) according to the sums, not the number of the balls.

1.1.4. Game: Digits, Watch out
■ Objective: to improve the students' knowledge of the digit order in an increasing number sequence, practice their perceptiveness and orientation in space and time, and improve certain motor abilities and physical skills.
■ Number of participants: any number
■ Props: an Eduball for each participant
■ Area: gymnasium or open air
■ Description: each student has a green or yellow ball with a number from 0 to 9. The teacher appoints catchers. The catchers are the students with balls with the digit "9" on them. The catchers' task is to tag students with the digit "8". After a student has been tagged, they pair up with the catcher and try to tag the student with the digit "7" together, etc. The game is over when all the digits have been tagged.
■ Variation: each student has a ball with a number from 0 to 9. They all have to tag a person with 16 their predecessor, e.g. a student with an "8" tries to tag the student with a "7" and a student with a "0" tries to tag a "9". After the predecessor has been tagged, the students pair up. The game is over when all students have formed the entire number sequence.

1.1.5. Game: Digit Order
■ Objective: to improve the recognition of digits in an increasing number sequence, develop the ability to work in a team, and practice certain motor skills. Number of participants: any number
■ Props: Eduballs
■ Area: gymnasium or open air
■ Description: The balls are strewn across one half of the gymnasium. The students pair up. Their task is to find all balls containing the digits mentioned by the teacher, to roll them to an indicated spot and form an increasing sequence. After all balls have been lined up, the students perform a special task that has been assigned to each number and repeat it according to the number, e.g. 1 – a 360°turn, 2 – 2 jumps, 3 – 3 squats, etc.

1.1.6. Game: Even and Odd Numbers
■ Objective: to practice the distinction between even and odd digits, and improve certain motor skills.
■ Number of participants: any number
■ Props: Eduballs
■ Area: gymnasium or open air
■ Description: Eduballs are strewn across the whole gymnasium. The students stand at the center circle. The teacher indicates places for even and odd numbers in the corners of the gymnasium. The

students move freely across the pitch. At the teacher's signal, they should put the balls into the indicated places according to the previous instructions.
■ Organizational remarks: the teams can compete against each other.
■ Variation: each student has a green or yellow ball. The teacher indicates bases for even and odd digits in the corners of the gymnasium. The students move across the pitch in a manner indicated by the teacher. At the teacher's signal, the students have to put their ball into the right corner according to the color of the ball and the digit on it. After they have completed the task, the students exchange balls.

1.1.7. Game: Mathematical Signs
■ Objective: to practice forming simple mathematical operations, develop the ability to work in a team, and improve certain motor skills.
■ Number of participants: any number
■ Props: an Eduball for each participant
■ Area: gymnasium or open air
■ Description: the students move about the pitch in different directions with Eduballs in four colors (yellow, green, blue with the signs +, –, = and red with the signs +, –, =) in their hands. At the teacher's signal, they regroup to form mathematical operations, e.g. 3+4=7; 9–8=1. If a student has not joined any group, they have to say an operation with the number or sign they have on their ball. After the task has been completed, the students exchange balls.

1.1.8. Game: Number Tag
■ Objective: to get familiar with the digit "5", learn mathematical operations with the number "5", develop orientation in space and time, and improve speed.
■ Number of participants: any number
■ Props: an Eduball for each participant
■ Area: gymnasium or open air
■ Description: each student has a ball in any color. The student who has the ball with the number "5" is the chaser. When the chaser tags a student, they exchange their balls and the tagged person becomes the chaser.
■ Variation: all students have green or yellow balls. The student who has the ball with the number "5" on it is the chaser. The person who gets tagged by the chaser, can gain protection if they say what the sum of their number and the number "5" is. They only have five seconds to do so. When someone says an incorrect sum or takes too much time, they become the chaser.

1.1.9. Game: Operations in The Air
■ Objective: to practice simple mathematical operations and improve orientation in space.
■ Number of participants: any number
■ Props: an Eduball for each participant
■ Area: gymnasium or open air
■ Description: the students move across the pitch, throwing Eduballs as high as they can. At the teacher's signal, they stop. The teacher lifts a ball with the addition (+) or subtraction (-) sign and sets a task, e.g. add the number you have on your ball to 2. After several tasks, the students exchange their balls. The tasks can also be solved in pairs. Then the students can check each other's answers and compare them, e.g. <, >, =.
■ Organizational remarks: the students performing these tasks practice addition and subtraction, as well as certain motor skills. The teacher evaluates the quality of their performance and how much

time they need to make a decision. There is a higher probability of the task being completed when students work in pairs.

1.1.10. Game: Subtractions

■ Objective: to practice calculations, develop orientation in space and time and improve running speed.
■ Number of participants: any even number
■ Props: Eduballs
■ Area: gymnasium or open air
■ Description: each student has an Eduball and moves freely across the gymnasium. At the signal, the teacher lifts a red ball with the minus sign (–). The students' task is to calculate the result of subtraction of the number they have on their ball from the number given by the teacher and saying the difference aloud. After having performed the task, the students exchange their balls. However, if the subtrahend is bigger than the minuend, the operation is infeasible.
■ Organizational remarks: The students performing this task practice subtracting up to 10. The teacher can also ask for the result individually, to find out which students have trouble with it.

1.2. Foreign Language Education

1.2.1. Game: Alphabet

■ Objective: to practice the order of the letters of the English alphabet, develop perceptiveness and focus and improve the ability to work in a team.
■ Number of participants: any even number
■ Props: Eduballs
■ Area: gymnasium or open air
■ Description: the students are divided into two teams (the green and yellow team). In the teams, the students line up with their hands at each other's shoulders, forming Eduball trains. The students set out on a journey to Eduball and to get to know all the letters of the English alphabet. The students move across the whole gymnasium singing the ABC song: "A - B - C - D - E - F - G; H - I - J - K - L - M - N - O - P; Q - R - S - T - U- V; W - X - Y and Z. Now I know my ABC. Next time won't you sing with me?". The yellow team collect yellow balls and the green team look for green balls.

1.2.2. Game: Catch Me if You Can

■ Objective: to enrich English vocabulary, get to know English names of animals, develop the ability to work in a team, and improve certain motor abilities and physical skills.
■ Number of participants: any number
■ Props: an Eduball for each participant
■ Area: gymnasium or open air
■ Description: each student has a green ball. Two students with balls with the letter C are chasers. They are the cats. The cats' task is to catch as many mice as possible. However, two dogs (students with balls with the letter D) and two pieces of cheese (students with yellow balls) will try to disturb them. Mice may find shelter from the cats for five seconds behind the dogs. The mice which have been tagged by cats go into the cage (e.g. sit on a bench). They can be, however, freed when tagged by the students with the yellow balls.
■ Organizational remarks: The teacher needs to often change roles performed by the students.

1.3. Interdisciplinary Education

1.3.1. Game: Multi-Tag

■ Objective: to practice knowledge in interdisciplinary educational areas (e.g. native language, mathematics, science, foreign languages), improve orientation in space and time and reaction speed.
■ Number of participants: any number
■ Props: an Eduball for each participant
■ Area: gymnasium or open air
■ Description: each student has a green or yellow ball. The teacher appoints three chasers with orange balls. The chasers have to tag the remaining students who can gain protection by saying a word in a given theme category (e.g. plants, animals, etc.) beginning with the letter on their ball.
■ Variation: each student has a green or yellow ball. The teacher appoints three chasers with an orange, red and blue ball. The orange chaser is assigned to English, the red one to the native language and the blue one to mathematics. The chasers have to tag the remaining students who can gain protection by saying a word in their native language or English beginning with a letter on their ball, or a mathematical operation where the digit on their ball is the result. When someone says an incorrect word, repeats it or takes too much time, they become the chaser.

1.3.2. Game: What time is it?

■ Objective: to teach reading the time on an analog clock, develop the ability to work in a group and improve certain motor skills.
■ Number of participants: any number
■ Props: Eduballs
■ Area: gymnasium or open air
■ Description: the teacher uses rubber rings to form the outline of an analogue clock. Together with the students, they place Eduballs with digits to mark the hours. In the center, the teacher places an orange ball. A red ball is placed for the hour hand and a blue ball for the minute hand. The students ask the question “What time is it?” and the teacher answers “It’s three o’clock”. The students now have to place the hands in the clock accordingly and perform a motor task as many times as is the number stated in the time, e.g. jump three times.

1.4. Native Language Education

1.4.1. Game: Letter Tag with The Letter “M”

■ Objective: to develop the skill of forming words beginning with the letter “m”, improve certain motor abilities and physical skills.
■ Number of participants: any number
■ Props: an Eduball for each participant
■ Area: gymnasium or open air
■ Description: each student has a yellow or green ball and moves freely around. Students who have a ball with the letter “m” are the chasers. Their task is to tag the rest of the students. A participant who has been tagged by the chaser can gain protection if they are able to say a word that begins with the letter “m”, e.g. “mother”. They only have 5 seconds to do so and cannot repeat what has already been said. When someone says an incorrect word, repeats it or takes too much time, they become the chaser.
■ Variation: The students can also gain protection by saying a word that only contains the letter “m”, e.g. “game”.

2. Supplementary results

Supplementary Table 1. Selected results on physical fitness before the experiment.

Variable	Experimental Class		Control Class	
	$\bar{x}$	*SD*	$\bar{x}$	*SD*
Run for 600 m [s]	236.26	23.78	265.95	36.21
Forward bend [cm]	–3.74	5.75	–4.24	5.69
Long jump from standstill [cm]	118.61	20.21	117.24	13.27
Run for 50 m [s]	12.38	1.25	12.45	0.73
Hand strength [kg]	12.65	3.08	13.29	2.63
Bent arm hang [s]	5.57	10.03	5.07	8.11
Sit-up from the lying position [number]	14.57	3.38	16.14	5.30
Shuttle run 4 x 10 m [s]	13.56	2.03	13.82	1.74
